# Tyrosine 48 Phosphorylation of Cytochrome *c* Alters Mitochondrial Respiration, ROS Production, and Apoptosis

**DOI:** 10.3390/biom16050632

**Published:** 2026-04-24

**Authors:** Paul T. Morse, Susanna Vuljaj, Nabil Yazdi, Matthew P. Zurek, Junmei Wan, Icksoo Lee, Asmita Vaishnav, Brian F.P. Edwards, Tasnim Arroum, Maik Hüttemann

**Affiliations:** 1 Center for Molecular Medicine and Genetics, Wayne State University, Detroit, MI 48201, USA; 2 Department of Biochemistry, Microbiology, and Immunology, Wayne State University, Detroit, MI 48201, USA; 3 College of Medicine, Dankook University, Cheonan-si 31116, Chungcheongnam-do, Republic of Korea

**Keywords:** mitochondria, hepatic, liver, cytochrome *c*, electron transport chain, apopotosis, reactive oxygen species, ROS, phosphorylation, Y48

## Abstract

Cytochrome *c* (Cyt*c*) tyrosine 48 (Y48) has been previously shown to be phosphorylated in bovine liver, and phosphomimetic substitution (Y48E) inhibits key functions of Cyt*c* in vitro, including respiration and apoptosis. In this study, we investigated the effect of Y48 modification in a double-knockout cell culture model that stably expressed either unphosphorylated wild-type (WT) Cyt*c*, control Y48F Cyt*c*, or phosphomimetic Y48E Cyt*c*. Our findings revealed that Y48E Cyt*c* caused partial inhibition of mitochondrial respiration in intact cells, which corresponded with lower mitochondrial membrane potentials (ΔΨ_m_) and reduced reactive oxygen species (ROS) production. When subjected to an oxygen–glucose deprivation/reoxygenation (OGD/R) model, which simulates ischemia/reperfusion injury, the Y48E phosphomimetic cell line showed lower ROS production compared to the unphosphorylated WT and Y48F Cyt*c* cell lines, the latter of which generated higher levels of ROS upon reoxygenation. As a result, the Y48E Cyt*c* cell line had significantly lower cell death rates when exposed to OGD/R, confirming the cytoprotective role of Y48 phosphorylation of Cyt*c*. In summary, our research indicates that the loss of Y48 phosphorylation in Cyt*c* during ischemia leads to reperfusion injury by driving maximum electron transport chain flow, hyperpolarization of ΔΨ_m_, bursts of ROS, and death of cells through apoptosis.

## 1. Introduction

Cytochrome *c* (Cyt*c*) is a small, globular, nuclear encoded mitochondrial protein with a covalently attached heme group [1]. It functions as a molecular switch between cellular respiration and intrinsic apoptosis. The function of Cyt*c* in cellular respiration involves the shuttling of electrons from complex III to complex IV (also known as cytochrome *c* oxidase or COX), which is the proposed rate-limiting step within the mitochondrial electron transport chain (ETC) [2,3]. COX then catalyzes the reduction of oxygen, the terminal electron acceptor, to water. In Cyt*c*’s other role, a pivotal moment for intrinsic apoptosis is when Cyt*c* is released from the mitochondria. When the cell is under stress, Cyt*c* is released from the mitochondria into the cytosol, where it binds to apoptotic protease-activating factor-1 (Apaf-1) and procaspase-9 to form the apoptosome, activating the caspase cascade, specifically procaspase-9 to caspase-9, which in turn activates procaspase-3 to caspase-3 [4,5,6,7]. Cyt*c* also has a variety of other cellular functions, such as detoxifying reactive oxygen species (ROS), redox-coupled protein import via the Erv1-Mia40 pathway, DNA damage response via protein phosphatase 2A, release of nucleophosmin, and supercomplex assembly [8,9,10,11,12]. Other pro-apoptotic functions of Cyt*c* are cardiolipin peroxidase activity and ROS formation via reduction of p66^Shc^ [13,14,15,16,17,18,19]. Given these important and diverse functions, Cyt*c* is strictly regulated by the cell via multiple mechanisms: ATP-mediated allosteric regulation, isoform expression, and post-translational modifications (PTMs) [3,20].

Cyt*c* has been found to be regulated by a wide variety of PTMs, including acetylation, carbonylation [21,22,23], deamidation [24], glycation [25,26,27,28], glycosylation [29,30], homocysteinylation [31,32,33], nitration [34,35,36,37,38], nitrosylation [39], phosphorylation, and sulfoxidation [40,41,42,43]. Specifically, seven phosphorylation sites on mammalian Cyt*c* have been identified as specific to certain tissues: Y97, T49 (numbering based on the mature peptide, which lacks the start methionine), and Y67 in the heart [44,45,46,47,48,49], Y48 in the liver [50,51,52,53], T28 and T58 in the kidney [54,55,56], and S47 in the brain [4,56,57]. These modifications strongly impact the functions of the protein, including respiration, apoptosis, cardiolipin peroxidase activity, and ROS scavenging capability. Specifically, as we and others have reported, these phosphorylations result in partial inhibition of respiration, which results in decreased mitochondrial membrane potential (ΔΨm) and decreased mitochondrial ROS production [13,14,50,54,55,58]. These phosphorylations also block the ability of Cyt*c* to initiate intrinsic apoptosis. As these phosphorylations have generally been found to be present under basal conditions and lost during ischemia when ATP is depleted, and kinases cannot operate but phosphatases are or become active, they appear to contribute to ischemia–reperfusion injury. Loss of Cyt*c* phosphorylations increases respiration, driving increased ΔΨm and therefore increased mitochondrial ROS production. Additionally, two tissue-specific acetylation sites have been characterized: K39 in ischemic skeletal muscle [1] and K53 in prostate cancer [59,60], both of which confer specific metabolic and apoptotic advantages to those particular pathophysiological conditions.

In our earlier study, we demonstrated that Y48 phosphorylation represents the primary PTM that occurs naturally in the liver of bovine animals [51]. Functional analysis on phosphorylated Y48 Cyt*c* indicated an inhibition in activity in reaction with purified COX due to Y48 phosphorylation [51]. Studies on phosphomimetic Y48E Cyt*c* have shown decreased activity in reaction with COX, abolished caspase-3 activity, decreased redox potential, and decreased cardiolipin peroxidase activity [50]. Using the evolved tRNA synthetase technique to replace tyrosine 48 with *p*-carboxy-methyl-L-phenylalanine has shown decreased supercomplex activity with increased activity in reaction with COX, decreased caspase-3 activity, and increased cardiolipin peroxidase activity [52]. Structural analyses have shown that Y48 phosphorylation modifies the heme environment by increasing intramolecular Cyt*c* dynamics, which contributes to decreased inhibition of SET/TAF-Iβ histone chaperone activity [9].

The objective of this study was to enhance our knowledge of the impact of Cyt*c* Y48 phosphorylation on multiple functions of Cyt*c*, including apoptosis and respiration, using purified Cyt*c* variants and cell lines stably expressing the Cyt*c* variants. Via mutagenesis, two Cyt*c* variants were obtained and studied in addition to wild-type (WT): unphosphorylatable control Tyr48Phe (Y48F) and phosphomimetic mutant Tyr48Glu (Y48E). In vitro, we found that phosphomimetic Y48E Cyt*c* showed decreased rate of oxidation, unchanged rate of reduction, and decreased heme stability. In cells stably expressing the various Cyt*c* variants, cells expressing the phosphomimetic Y48E Cyt*c* showed decreased respiration, decreased ΔΨm, ROS, and ATP levels, decreased cell death after exposure to cellular stressors, and blunted responses to OGD/R. Altogether, these findings support the hypothesis that Cyt*c* phosphorylation is protective against ischemia–reperfusion injury and that the loss of Cyt*c* phosphorylation during ischemia sensitizes the cell to reperfusion injury.

## 2. Methods

All chemicals and reagents were purchased from MilliporeSigma (Burlington, MA, USA) unless otherwise specified.

### 2.1. Plasmid Design and Purification

The codon corresponding to Y48 of the somatic rodent Cyt*c* (WT) cDNA cloned into either the bacterial expression plasmid pLW01 or eukaryotic pBABE-puromycin expression plasmid (Addgene, Cambridge, MA, USA) was mutated to phenylalanine as an additional control (Y48F), which cannot be phosphorylated, or glutamate (Y48E) as a phosphomimetic replacement through the utilization of the QuikChange II site-directed mutagenesis kit (Agilent Technologies, Santa Clara, CA, USA). The pLW01 bacterial expression plasmid also includes the cDNA encoding *S. cerevisiae* heme lyase (CYC3), which bacteria lack, in order to covalently incorporate the heme group into Cyt*c* [61]. The following mutagenesis primers were used to mutate and amplify the plasmid: Y48F Forward: 5′-GCT GGA TTC TCT TTC ACA GAT GCC-3′, Reverse: 5′-GGC ATC TGT GAA AGA GAA TCC AGC-3′; Y48E Forward: 5′-GCT GGA TTC TCT GAA ACA GAT GCC-3′, Reverse: 5′-GGC ATC TGT TTC AGA GAA TCC AGC-3′. The PCR products underwent a one-hour incubation at 37 °C with Dpnl endonuclease from the QuikChange II site-directed mutagenesis kit to digest methylated parental DNA while preserving the newly synthesized DNA. The mutant plasmids were then transformed into X10-Gold ultracompetent cells (QuikChange II site-directed mutagenesis kit) according to the manufacturer’s protocol. Individual bacterial colonies were selected for each mutant, and all plasmids from overnight cultures were purified using QIAprep spin miniprep kit (#27106, Qiagen, Valencia, CA, USA). Final plasmid purity and concentration were assessed using a Nanodrop 1000 spectrophotometer. Successful Cyt*c* mutations were confirmed by sequencing.

### 2.2. Recombinant Protein Expression and Purification

The Cyt*c* variants were overexpressed by transforming the Cyt*c* mutant pLW01 plasmids into chemically competent *Escherichia coli* strain CD41(DE3) cells (Biosearch Technologies, Petaluma, CA, USA) via heat shock. Selected colonies were inoculated into 10 mL of terrific broth containing 100 μg/mL carbenicillin and grown overnight at 37 °C. These overnight cultures were then inoculated and grown in several flasks containing a total of 5 L of TB medium containing 100 μg/mL carbenicillin until an OD_600_ of 0.8 or greater was achieved. Cyt*c* expression was induced by adding 100 mM isopropyl-β-D-thiogalactoside. The cells were harvested after overnight culture via centrifugation at 1900 RPM (Shovell SS-34, Thermo Ficher Scientific, Waltham, MA, USA) for 55 min at 4 °C. Collected bacterial pellets were lysed, and Cyt*c* was purified using modified ion exchange chromatography conditions for the DE52 anion exchange column (20 mM phosphate buffer, pH 7.5, 4.0 mS/cm conductivity), followed by CM52 cation exchange (30 mM phosphate buffer, pH 6.5, 6.0 mS/cm conductivity), which were adapted from Mahapatra et al. [54]. The protein was eluted using a high salt elution buffer (40 mM Kpi, 0.5 M NaCl), desalted, and concentrated using Amicon Ultra 15 3 kDa centrifugal filter units (Millipore, Billerica, MA, USA) before being stored at −80 °C as previously described in [1,48,49,50,54].

### 2.3. UV-Vis Spectra

Cyt*c* purified protein concentration was determined using a Jasco V-570 double-beam spectrophotometer (2 nm bandwidth, 200 nm/min scanning speed). The Cyt*c* variants were either oxidized with potassium ferricyanide (K_3_Fe(CN)_6_) or reduced with sodium dithionite (Na_2_S_2_O_4_) and then desalted using Sephadex G-25 NAP5 columns (#17-0853-02, GE Healthcare, Piscataway, NJ, USA). Cyt*c* concentration was calculated using differential spectra at 550 nm, obtained by subtracting the oxidized form from the reduced form, and applying the formula [Cyt*c*] = (A_550 reduced_ − A_550 oxidized_)/(19.6 mM/cm × 1 cm) × dilution factor [55]. The presented reduced Cyt*c* UV-Vis spectra were measured at 25 μM Cyt*c*. Cyt*c* purity was confirmed by Coomassie blue staining on a 10% Tris-tricine SDS-PAGE gel, as previously described in [1,48,49,50,54].

### 2.4. Rate of Oxidation

The technique utilized for determining the rate of oxidation that was employed has been previously reported [54,62]. Initially, variants of Cyt*c* were fully reduced using Na_2_S_2_O_4_, and the reductant was separated from the recombinant proteins via NAP-5 columns. Cyt*c* (15 µM) was mixed with H_2_O_2_ (100 µM), the oxidizing agent, in 0.2 M Tris-Cl, pH 7.0. The reduction in the absorption peak at 550 nm was measured every 10 s after adding H_2_O_2_. The concentration of oxidized Cyt*c* was calculated using the above spectrophotometric method, which was then used to calculate the rate of Cyt*c* oxidation, expressed in μM/s, as previously described in [1,48,49,50,54].

### 2.5. Rate of Reduction

Variants of Cyt*c* were fully oxidized using K_3_Fe(CN)_6_, and the oxidant was separated away from the recombinant proteins via NAP-5 columns (#17-0853-02, GE Healthcare, Piscataway, NJ, USA) as described [54]. Cyt*c* (10 μM) was mixed with hypoxanthine (100 μM) and catalase (14.2 nm) in 1× PBS. The reaction was initiated with the addition of 181.5 nM xanthine oxidase, which generated superoxide as the reductant. The increase in absorption peak at 550 nm was measured every 15 s after adding xanthine oxidase. Superoxide dismutase (925 nM) was also added as a negative control in certain trials, as it detoxifies the superoxide before it can react with oxidized Cyt*c* [59]. The concentration of reduced Cyt*c* was calculated using the above spectrophotometric method, as above, and rates are reported as μM/s as previously described in [1,48,49,50,54].

### 2.6. Heme Degradation

The degradation of the covalently attached heme group of Cyt*c* was analyzed by monitoring the dissipation of the Soret band at 408 nm [63]. Initially, variants of Cyt*c* were fully oxidized using K_3_Fe(CN)_6_, and the oxidant was separated away from the recombinant proteins via NAP-5 columns. Cyt*c* (5 μM) in 50 mM phosphate buffer, pH 6.1, was prepared. The degradation of the heme group was initiated by adding a high concentration of H_2_O_2_ (3 mM). The loss in absorbance at 408 nm was measured at 60, 200, 400, 600, and 800 s. The heme degradation is reported as a percent change comparing the absorbance at 408 nm to the baseline absorbance at 408 nm, as previously described in [1,48,49,50,54].

### 2.7. Establishing Cell Lines with Stable Expression of Cytc Variants

The WT, Y48E, and Y48F Cytc pBABE expression constructs created above were introduced into Cyt*c* double-knockout mouse lung fibroblasts (a kind gift from Dr. Carlos Moraes, University of Miami, Coral Gables, FL, USA) via stable transfection, employing Transfast transfection reagent (Promega, Madison, WI, USA) at a 1:1 ratio of transfection reagent to DNA, following the manufacturer’s protocol. As a negative control, cells were also transfected with a pBABE plasmid that did not contain the sequence for Cyt*c*, resulting in an empty vector (EV) cell line. The transfected cells underwent cultivation in DMEM supplemented with 10% FBS (Sigma-Aldrich, St. Louis, MO, USA), 1% Pen/Strep, 1 mM sodium pyruvate, and 50 µg/mL uridine in selection media containing 3 µg/mL puromycin at 37 °C in 5% CO_2_. Positive clones were selected, and their populations were expanded in DMEM supplemented with 10% FBS and 1% Pen/Strep. Experiments were performed with cells cultured in 10% FBS, 1 mM sodium pyruvate, and 1% Pen/Strep at 37 °C with 5% CO_2_ unless otherwise stated.

### 2.8. Gel Electrophoresis and Western Blotting

Cells expressing the recombinant Cyt*c* variants were lysed using 100 μL RIPA lysis buffer (150 mM NaCl, 5 mM EDTA, 50 mM Tris, 1% NP-40, 0.5% sodium deoxycholate, 0.1% SDS, pH 8.0) supplemented with protease inhibitor cocktail (#P8340, MilliporeSigma) to prevent protein degradation of the lysate. Mixtures were sonicated for 30 s total in 5 s pulse intervals and centrifuged at 16,900× *g* for 20 min at 4 °C to collect cellular debris. Western analysis following SDS polyacrylamide gel electrophoresis (SDS-PAGE) of Cyt*c* was then carried out. For each cell line, 50 µg of cell lysate was loaded onto a 10% Tris-tricine SDS-PAGE gel, along with anode (200 mM Tris-base, pH 8.9) and cathode (100 mM Tris-base, 100 mM Tricine, 0.1% SDS, pH 8.25) running buffers. The gel was then transferred onto an immuno-blot PVDF membrane (#1620177, Bio-Rad, Hercules, CA, USA) using a Trans-Blot SD semi-dry apparatus (#1703940; Bio-Rad) at 75 mA for 15 min and blocked in 5% non-fat dry milk at room temperature for 1 h. The membrane was then incubated overnight at 4 °C with either 1:4000 mouse anti-Cyt*c* (#556443, BD Pharmingen, San Jose, CA, USA) or 1:8000 mouse anti-GAPDH (#60004-1-Ig, Proteintech, Rosemont, IL, USA) in 5% non-fat dry milk. The next day, the membrane was incubated for 2 h in 1:10,000 sheep anti-mouse IgG conjugated horseradish peroxidase secondary antibody (#NA931V, GE Healthcare, Chicago, IL, USA) in 5% non-fat dry milk at room temperature. The blots were visualized using Pierce ECL Western blotting substrate (#32106, Thermo Fisher Scientific, Waltham, MA, USA) after a 2 min incubation. Signal intensities were quantified using ImageJ v1.52a with the Cyt*c*/GAPDH signal intensity normalized to WT, which was set at 1.0.

### 2.9. Cell Growth Rate

A total of 30,000 cells per well were seeded in a 0.1% gelatin-coated Costar 12-well plate. After every 24 h, three wells were harvested via trypsin, and the cell count was determined using a Moxi Z automated cell counter (#MXZ000, Orflo, Ketchum, ID, USA).

### 2.10. Oxygen Consumption via Seahorse Assay

The oxygen consumption rates (OCR) and extracellular acidification rates (ECAR) of the stable cell lines expressing WT, Y48E, Y48F, and EV were assessed via mitochondrial stress test. Cells were seeded at a density of 20,000 cells per well in a 0.1% gelatin-coated XF24 plate (#100777-004, Agilent) with 250 µL/well of growth media and incubated at 37 °C, 5% CO_2_ for overnight. The following day, the growth medium was replaced with 675 µL of Seahorse media. This was made by dissolving 4.15 g of DMEM powder (#D5030, Millipore Sigma) in 500 mL of ddH_2_O (pH 7.4), supplemented with 10 mM glucose and 10 mM sodium pyruvate without FBS or phenol red, and filtering it with a sterile filter (#431097, Corning Incorporated, Corning, NY, USA). The cells were incubated in a CO_2_-free incubator for 1 h before measurements were taken. Next, the basal OCR, basal ECAR, non-mitochondrial respiration, proton leak, ATP-coupled respiration, maximal respiration, and spare respiratory capacity were measured using an XFe24 Seahorse extracellular flux analyzer (Seahorse Biosciences, North Billerica, MA, USA). A mitochondrial stress test was conducted by injecting oligomycin (1 μM), carbonyl cyanide chlorophenylhydrazone (CCCP, 2.5 μM), and rotenone/antimycin A (1 μM) sequentially, according to the manufacturer’s protocol. OCR data is reported as pmol O_2_/min, and ECAR is reported as mpH/min.

### 2.11. Membrane Potential

To measure relative mitochondrial membrane potential (ΔΨ_m_) changes, the JC-10 probe (#ENZ-52305, Enzo Life Sciences, Farmingdale, NY, USA) was used. Cells were seeded at a density of 20,000 cells/well onto a black 96-well plate (#CLS3603, Corning) coated with 0.1% gelatin and cultured overnight. The cells were then incubated in FBS-free, phenol red-free DMEM cell culture medium supplemented with 3 μM JC-10 for 30 min at 37 °C, 5% CO_2_. After incubation, the cells were washed twice with 1× PBS. The green fluorescence (excitation 485 nm/emission 527 nm) and red fluorescence (excitation 485 nm/emission 590 nm) of the cells were measured using a Fluoroskan Ascent FL plate reader (Thermo Fisher Scientific). ΔΨ_m_ was calculated as the ratio of red/green fluorescence as previously described in [1,48,49,54].

### 2.12. Reactive Oxygen Species Production

To measure mitochondrial ROS production, the MitoSOX probe (#M36008, Invitrogen, Carlsbad, CA, USA) was used. Cells were seeded at a density of 20,000 cells/well onto a black 96-well plate (#CLS3603, Corning) coated with 0.1% gelatin and cultured overnight. The cells were then incubated in FBS-free, phenol red-free DMEM cell culture medium supplemented with 5 μM MitoSOX for 30 min at 37 °C, 5% CO_2_. Cells were washed with 1× PBS, and their fluorescence (excitation 510 nm/emission 580 nm) was assessed using a Synergy H1 plate reader (BioTek, Winooski, VT, USA). Data is reported as a percentage change compared to WT, as previously described in [1,48,49,54].

### 2.13. ATP Levels

Cells were seeded at 1,000,000 cells/dish onto 10 cm cell culture dishes (#664160, Greiner Bio-One, Frickenhausen, Germany) and cultured overnight. Within 90 s, cells were collected by scraping and briefly centrifuged, with the resulting pellets flash frozen via liquid nitrogen. Flash-frozen cell pellets were boiled for 2 min in 300 μL boiling buffer (100 mM Tris-Cl, 4 mM EDTA, pH 7.75), followed by sonication on ice. The resulting lysates were diluted 300-fold, and the ATP concentration of 40 μL of the diluted lysate was measured using the ATP Bioluminescence assay kit HS II (#1169970900, Roche, Indianapolis, IN, USA), according to the manufacturer’s instructions using an Optocomp 1 luminometer (MGM Instruments, Hamden, CT, USA) as previously described in [1,48,49,54].

### 2.14. Flow Cytometry Apoptosis Assay

The levels of cell death in stable cell lines expressing WT, Y48E, Y48F, and EV were measured using annexin V/propidium iodide (PI) staining and assessed with flow cytometry. Cells were seeded at 1,000,000 cells/dish onto 10 cm cell culture dishes (#664160, Greiner Bio-One, Frickenhausen, Germany) and cultured overnight. The cells were then treated with either H_2_O_2_ (400 μM for 16 h) or staurosporine (1 μM for 5 h). Live and dead cells were harvested and washed twice with 1× PBS. A total of 1,000,000 cells were resuspended in 1 mL 1× binding buffer (BD Pharmingen, San Diego, CA, USA). A total of 450 μL cell suspension was incubated with 6 μL annexin V-FITC (BD Pharmingen) and 6 μL PI (BD Pharmingen) at room temperature in the dark for 15 min. After incubation, reagents were diluted with 3 mL 1× binding buffer and counted on a CyFlow Space flow cytometer (Sysmex America, Inc., Lincolnshire, IL, USA). Results were analyzed using FCS Express 7 software (De Novo Software, Glendale, CA, USA). Data are expressed as a percentage of cells as previously described in [1,48,49].

### 2.15. Membrane Potential and Reactive Oxygen Species Production After Oxygen–Glucose Deprivation/Reoxygenation (OGD/R)

To simulate ischemia/reperfusion conditions, stable cell lines expressing Cyt*c* variants underwent transient oxygen–glucose deprivation/reoxygenation (OGD/R). Cells were seeded and cultured according to the membrane potential or reactive oxygen species assays as described above. The medium was then replaced with glucose-free, FBS-free, phenol red-free DMEM (#A1443001, Gibco, Waltham, MA, USA) that was bubbled with 95% N_2_ and 5% CO_2_ to remove oxygen. The cells were then incubated under 1% O_2_ and 5% CO_2_ in a hypoxic chamber, which was controlled by ProOx 110 oxygen and ProCO_2_ 120 carbon dioxide controllers (BioSpherix; Redfield, NY, USA) for 90 min at 37 °C. Following oxygen–glucose deprivation, the cells were reoxygenated for 30 min with glucose-containing, FBS-free, phenol red-free DMEM culture medium (#31053028, Gibco) supplemented with either JC-10 or MitoSOX. Control plates were maintained in normoxic conditions with regular growth media supplemented with FBS. The JC-10 and MitoSOX experiments were then carried out as described above, as previously described in [1,48,49].

### 2.16. Statistical Analyses

Data shown represent the mean, and error bars represent the standard deviation. For most assays, statistical analyses for the data were performed for statistical significance using one-way ANOVA comparing the mean of the WT column with the mean of every other column, followed by a post hoc Dunnett test using R version 4.5.0 via RStudio. The packages ggplot2, readxl, ggthemes, ggprism, dplyr, tidyr, and multcomp were used. For heme degradation, statistical significance was calculated using one-way ANOVA with post hoc Dunnett test as described above, specifically on the 800 s condition. For annexin V/propidium iodide experiments, statistical significance was calculated using one-way ANOVA with post hoc Dunnett test as described above on total cell death (PI+ cells, annexin V+ cells, and annexin V+/PI+ cells combined). For OGD/R experiments, statistical significance within the hypoxia and normoxia groups was calculated using one-way ANOVA with post hoc Dunnett test as described above; however, a student’s two-tailed *t*-test assuming equal variance was used to compare the hypoxia and normoxia conditions within each specific Cyt*c* variant. *p*-values are indicated in the figures.

## 3. Results

### 3.1. Purification of Recombinant Cytc Proteins via Bacterial Overexpression

**Figure 1:**
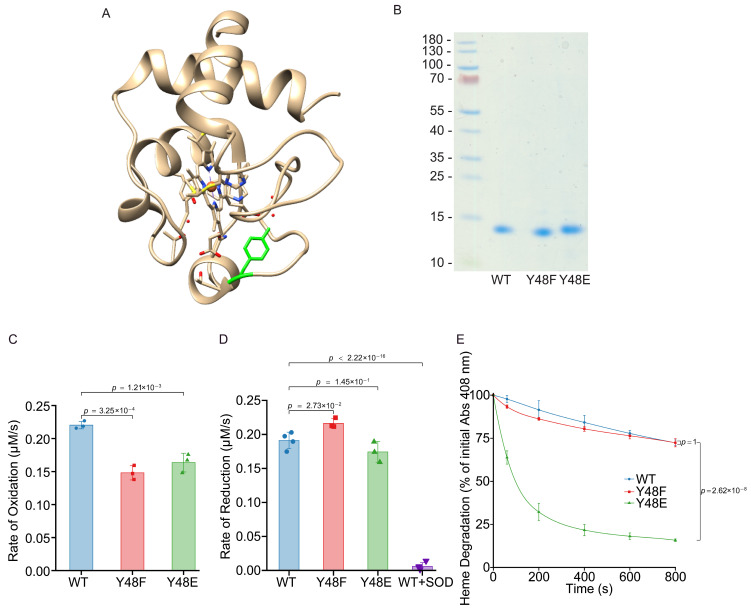
(**A**) Diagram of Cyt*c* (tan) with Y48 (green) labeled based on rodent WT Cyt*c* crystal structure (5C0Z.pdb) processed with the program Chimera (version 1.16). (**B**) Representative Coomassie blue stained 10% Tris-tricine SDS-PAGE gel showing the purity of recombinant WT, Y48F, and Y48E Cyt*c* proteins. (**C**) Initial rate of oxidation of reduced recombinant Cyt*c* variant proteins in the reaction with 100 µM H_2_O_2_ (*n* = 3). (**D**) Initial rate of reduction of oxidized recombinant Cyt*c* variant proteins using superoxide generated xanthine oxidase and 100 μM hypoxanthine (*n* = 3–4). (**E**) Heme degradation of recombinant Cyt*c* variant proteins in the presence of high excess concentrations of H_2_O_2_ (3 mM, *n* = 3).

To study the effect of Y48 phosphorylation in vitro, we generated Y48E phosphomimetic Cyt*c* (Figure 1A). Phosphomimetic amino acid replacement can functionally mimic protein phosphorylation and be used to study the functional effects of fully phosphorylated proteins. We and others have applied this approach to rodent Cyt*c* (somatic mouse and rat Cyt*c* have identical amino acid sequences) [8] and other proteins [64] by replacing phosphorylation sites within Cyt*c* with the negatively charged amino acid glutamate, which produced similar functional effects as in vivo-phosphorylated Cyt*c*. We also generated an additional control, Y48F, which cannot be phosphorylated. Using an *E. coli* overexpression system with our purification protocol, we generated the recombinant Cyt*c* variants as shown via Coomassie blue-stained gel (Figure 1B). The UV-Vis spectra were similar as previously reported [50], demonstrating correct folding and successful incorporation of the heme group.

### 3.2. Phosphomimetic Y48E Cytc Shows Decreased Rate of Oxidation, Unchanged Rate of Reduction, and Is Less Resistant to Degradation of the Heme Group

Cyt*c* functions as a scavenger for both hydrogen peroxide and superoxide [58,65]. Considering the antioxidant role of Cyt*c*, we determined the rate of Cyt*c* oxidation by hydrogen peroxide and the rate of Cyt*c* reduction by superoxide [66]. To determine the rate of Cyt*c* oxidation, recombinant Cyt*c* variants were fully reduced with sodium dithionite. The ferro-Cyt*c* variants were oxidized in the presence of 50 µM H_2_O_2_. Phosphomimetic Y48E Cyt*c* displayed a 26% reduced oxidation rate compared to the WT (Figure 1C). To determine the rate of Cyt*c* reduction, recombinant Cyt*c* variants were fully oxidized with potassium ferricyanide. The ferri-Cyt*c* variants were reduced in the presence of superoxide generated by the hypoxanthine/xanthine oxidase reaction system. Phosphomimetic Y48E Cyt*c* did not show a statistically significant difference in rate of reduction compared to WT (Figure 1D). High concentrations of ROS are known to cause Cyt*c* to lose its ability to function. The measurement of heme degradation was used to test its stability when adding a large excess of 100 mM H_2_O_2_. Phosphomimetic Y48E Cyt*c* showed 78% increased heme degradation when compared to WT (Figure 1E).

### 3.3. Expression of Recombinant Cytc Proteins in a Cytc Double-Knockout Cell Line

**Figure 2:**
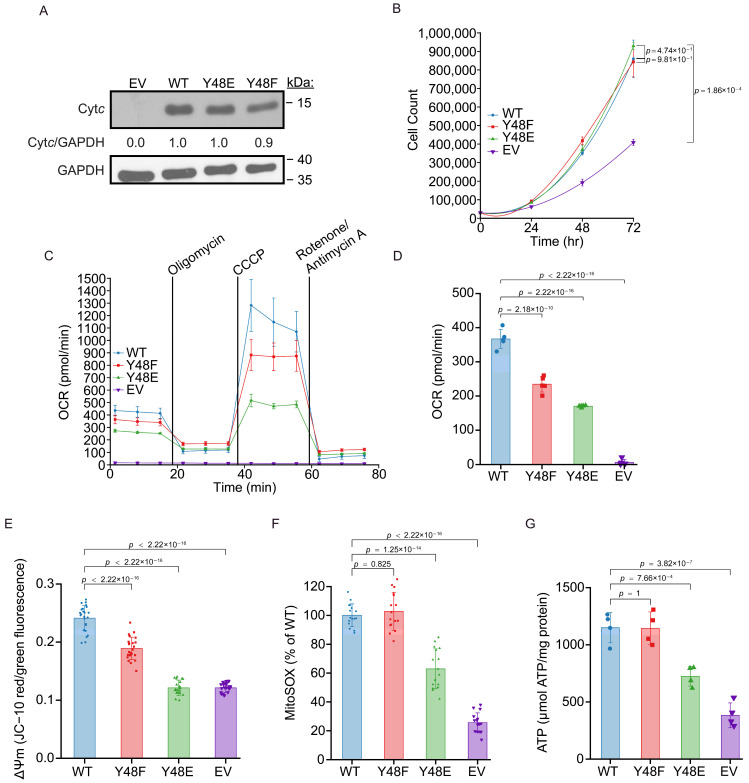
(**A**) Representative Western blot of Cyt*c* double-knockout cells transfected with recombinant Cyt*c* variants with GAPDH as a loading control. Protein intensities normalized to GAPDH are indicated relative to WT. (**B**) Cellular growth rate over 72 h of cell lines stably expressing recombinant Cyt*c* variants (*n* = 3). (**C**) Mitochondrial stress test of cell lines stably expressing recombinant Cyt*c* variants (*n* = 5). (**D**) Basal oxygen consumption (OCR) of cell lines stably expressing recombinant Cyt*c* variants derived from mitochondrial stress test (*n* = 5). (**E**) Mitochondrial membrane potential (ΔΨm) of cell lines stably expressing recombinant Cyt*c* variants (*n* = 24). (**F**) Mitochondrial ROS production of cell lines stably expressing recombinant Cyt*c* variants (*n* = 16). (**G**) ATP levels of cell lines stably expressing recombinant Cyt*c* variants (*n* = 4). The original gel electrophoresis images can be found in the Supplementary Materials.

To investigate the functional effects of the Y48E phosphomimetic substitution of Cyt*c* within a cell culture model, we employed a mutagenesis technique to create cell lines that stably expressed WT, Y48F, and Y48E Cyt*c,* as well as an empty vector (EV) cell line that does not express Cyt*c*. We established these Cyt*c* variants by transfecting recombinant plasmids into a Cyt*c* double-knockout mouse lung fibroblast cell line, where both the rodent testes and somatic isoforms of Cyt*c* were knocked out. In order to compare the effects of Cyt*c* mutation, clones were selected that expressed WT, Y48F, and Y48E Cyt*c* at similar levels, as confirmed by Western blot analysis (Figure 2A). In addition, we observed that there is no difference in cell growth rate of the cell lines stably expressing WT, Y48F, or Y48E Cyt*c* (Figure 2B).

### 3.4. Phosphomimetic Y48E Cytc Reduces Mitochondrial Respiration in Intact Cells

The two primary energy metabolism pathways that support cellular life are glycolysis and mitochondrial oxidative phosphorylation. Alterations in Cyt*c* impact overall cellular respiration due to the reaction between Cyt*c* and COX being the proposed rate-limiting step of the ETC (reviewed in [3]). Using a Seahorse bioanalyzer, the intact cell oxygen consumption rate (OCR) of the four cell lines was measured via mitochondrial stress test (Figure 2C). The cells expressing the phosphomimetic Y48E Cyt*c* showed 54% reduced basal OCR compared to the WT (Figure 2D).

### 3.5. Phosphomimetic Y48E Cytc Shows Lower Mitochondrial Membrane Potential, Decreased Reactive Oxygen Species Production, and Lower ATP Levels in Intact Cells

It is known that there is a relationship between OCR, ΔΨ_m_, and ROS production. Given that the cells expressing phosphomimetic Y48E Cyt*c* showed reduced OCR, we hypothesized that there should be concomitant decreases in ΔΨ_m_ and ROS production. ΔΨ_m_ was measured using the ratiometric probe JC-10. The red (high ΔΨ_m_) to green (low ΔΨ_m_) fluorescence ratio, which is an indicator of ΔΨ_m_, was 50% decreased in the cells expressing the phosphomimetic Y48E Cyt*c* compared to the WT (Figure 2E). Similarly, mitochondrial ROS production, measured using the MitoSOX probe, was 37% decreased in the cells expressing the phosphomimetic Y48E Cyt*c* compared to the WT (Figure 2F). Maintaining optimal, intermediate ΔΨ_m_ allows the cell to produce near maximal ATP while minimizing ROS production. Given this, the ATP production, assessed using a luciferase assay, in the cells expressing the phosphomimetic Y48E Cyt*c* were 37% decreased compared to the WT (Figure 2G). Additionally, cells expressing phosphomimetic Y48E Cyt*c* showed increased non-mitochondrial respiration and decreased proton leak, ATP-coupled respiration, maximal respiration, and spare respiratory capacity compared to the WT (Supplementary Figure S1).

### 3.6. Phosphomimetic Y48E Cytc Reduces Cell Death in Intact Cells

**Figure 3:**
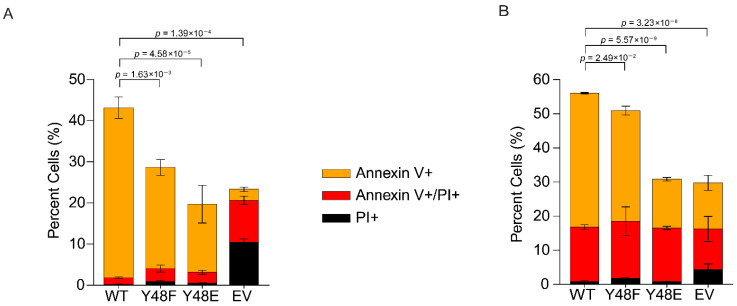
(**A**) Cell death rates of cell lines stably expressing recombinant Cyt*c* variants measured via flow cytometry after annexin V/propidium iodide (PI) staining of cells exposed to 400 μM H_2_O_2_ for 16 h (*n* = 3). (**B**) Cell death rates of cell lines stably expressing recombinant Cyt*c* variants measured via flow cytometry after annexin V/propidium iodide staining of cells exposed to 1 μM staurosporine for 5 h (*n* = 3). Orange—annexin V-positive cells, early apoptotic cells; red—annexin V- and PI-positive cells, late apoptotic cells; black—PI-positive cells, necrotic cells.

We used annexin V/propidium iodide (PI) staining followed by flow cytometry analysis to evaluate cell death. The cells were subjected to 400 µM H_2_O_2_ for 16 h or 1 µM staurosporine for 5 h. In both conditions, cells expressing the phosphomimetic Y48E Cyt*c* showed decreases in total cell death. In the H_2_O_2_ condition, 20% of cells expressing the phosphomimetic Y48E Cyt*c* underwent cell death compared to 43.2% of the WT (Figure 3A). In the staurosporine condition, 31% of cells expressing the phosphomimetic Y48E Cyt*c* underwent cell death compared to 56% of the WT (Figure 3B).

### 3.7. Phosphomimetic Y48E Cytc Reduces Mitochondrial Membrane Potential and Reactive Oxygen Species Production After Oxygen–Glucose Deprivation/Reoxygenation (OGD/R)

**Figure 4:**
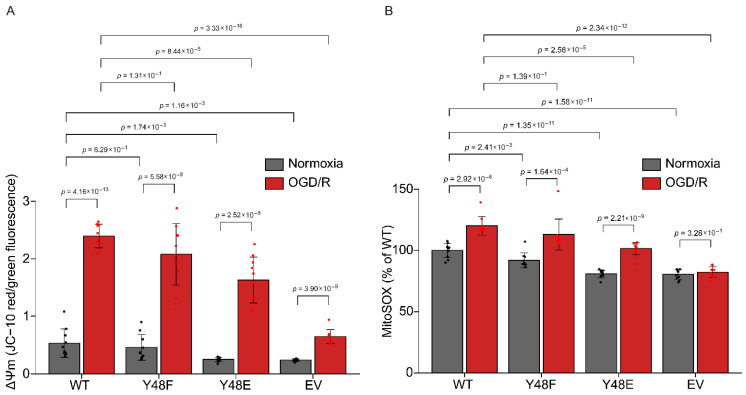
(**A**) Relative changes in ΔΨm of cell lines stably expressing recombinant Cyt*c* variants measured at normoxia (gray bars) or after 90 min of oxygen–glucose deprivation followed by 30 min of reoxygenation (red bars) (*n* = 10). (**B**) Mitochondrial ROS of cell lines stably expressing recombinant Cyt*c* variants measured at normoxia (gray bars) or after 90 min of oxygen–glucose deprivation followed by 30 min of reoxygenation (red bars) (*n* = 10).

Under normal conditions, ROS levels in cells are relatively low, but they can significantly increase under stress, such as during reperfusion following ischemia [67]. To evaluate the impact of the Y48E modification on cellular stress, such as ischemia–reperfusion injury, we used an oxygen–glucose deprivation/reoxygenation (OGD/R) model to simulate ischemia–reperfusion injury in cultured cells. Our stably transfected cell lines were exposed to 1% oxygen in glucose-free media for 90 min, followed by simulated reperfusion for 30 min, in which the complete culture media was supplemented with the probe JC-10 for ΔΨ_m_ analysis or MitoSOX for mitochondrial ROS production. Excluding EV cells, which do not contain Cyt*c* and thus have defective mitochondria, the cells expressing the phosphomimetic Y48E Cyt*c* showed the lowest levels of ΔΨ_m_ after OGD/R compared to the other Cyt*c* variants (Figure 4A). Similar findings were observed after OGD/R for MitoSOX. The cells expressing phosphomimetic Y48E Cyt*c* showed the lowest mitochondrial ROS production after OGD/R, excluding EV (Figure 4B). These results suggest a protective role of Y48E phosphomimetic replacement and thus Y48 phosphorylation as a possible target to ameliorate I/R injury.

## 4. Discussion

Under normal physiological conditions, tissue-specific phosphorylations of Cyt*c* act as a biological brake on mitochondrial activity, slowing down flux in the ETC and suppressing apoptosis to a different extent depending on the site. These modifications serve as metabolic regulators, optimizing tissue performance and enhancing resilience against everyday stressors. The loss of these phosphorylations during acute stress conditions, such as ischemia with loss of mitochondrial Ca^2+^ homeostasis due to rapid depletion of ATP, leads to activation of phosphatases and, thus, removal of the protective phosphorylations. In turn, when oxygen flux is restored to the tissue, the ETC works at a maximal capacity. This results in reperfusion injury due to ΔΨ_m_ hyperpolarization driving increased ETC activity, reverse electron transfer, and ROS bursts (reviewed in [68]) with another deleterious effect being the sensitization of the tissue to apoptosis [4,57].

Y48 is evolutionarily conserved across species [69], suggesting that it is crucial for Cyt*c* function and/or regulation. We have previously identified that Cyt*c* isolated from normal bovine liver tissue was Y48 phosphorylated and showed that the phosphomimetic substitution of Cyt*c* Y48 decreases respiration and binding to cardiolipin and abolishes its ability to trigger downstream caspase activation [50]. Y48 phosphorylation of Cyt*c* is an important modification that regulates COX and caspase-3 activity, as was shown in vitro [50]. Changes in respiration may be explained through both steric interference when Cyt*c* binds to the *bc*_1_ complex and COX, as well as the redox potential. The WT, Y48F, and Y48E redox potentials are about 237 mV, 207 mV, and 192 mV, respectively [50]. While the WT redox potential is between that of *bc*_1_ complex and COX, both mutants move closer to the midpoint redox potential of the *bc*_1_ complex. This could imply that electron transfer from *bc*_1_ to Cyt*c* is less favored, also slowing down the ETC at this step. However, the redox potential change cannot account for the decreased rate seen in the reaction with COX, which should make electron transfer more favorable [50]. This suggests that for the Y48E mutant, structural alterations, steric interference, and electrostatic repulsion from the negatively charged Cyt*c* binding site on COX, due to the introduction of the negatively charged glutamate residue, may cause the inhibition. The fact that the control Y48F mutant does not always behave like the WT and sometimes shows functional changes that reside between WT and Y48E Cyt*c*, for example, in live cell respiration, relative ΔΨ_m_ levels, and cell death experiments following H_2_O_2_ and staurosporine challenge, suggests that even the absence of the hydroxy group causes functional changes. This highlights the importance of this fully conserved residue for it to function optimally.

In this study, we expanded our investigation by characterizing the effects of phosphomimetic Y48E replacement on ROS scavenging and redox properties in vitro and by using a stable cell culture model expressing WT, Y48F, or Y48E Cyt*c*. Rodents possess two active Cyt*c* isoforms, somatic and testes. The single active human form of Cyt*c* possesses features of both rodent isoforms. In rodents, knocking out the somatic Cyt*c* isoform induces expression of the other isoform, ensuring metabolic competence [70]. Therefore, we employed a double knockout model where both the somatic and testes isoforms were knocked out and transfected this cell line with the recombinant Cyt*c* variants to mimic the effects of Y48 phosphorylation. Our data here using intact cells agrees with the previously reported findings regarding Y48 phosphorylation of Cyt*c*, inhibiting the respiratory and apoptotic capabilities of Cyt*c*. In cells expressing phosphomimetic Y48E Cyt*c*, there is decreased mitochondrial respiration, ΔΨ_m_, and ROS production. Furthermore, in cells expressing phosphomimetic Y48E Cyt*c*, there were lower levels of total cell death after exposure to either H_2_O_2_ or staurosporine.

A limitation of our study is that the WT, Y48F, and Y48E cell lines do not express the exact same amount of Cyt*c*, which could impact some of the results shown with the live cells. While WT and Y48E match well regarding their expression levels, the cell line expressing Y48F shows about 10% less expression. We established multiple clones for the two mutants and picked for each the clone that showed expression levels that were closest to the WT. In addition, it would be interesting to know the Cyt*c* expression levels of the parental cell line that was used to generate the Cyt*c* double-knockout cell line, which is not available to us, and to compare it to the cell lines generated here.

In addition to its well-established function in the ETC and as part of the apoptosome, Cyt*c* also functions in the nucleus, related to DNA damage response and histone regulation [9,11]. The authors reported that Cytc interacts with histone chaperones SET/TAF-Iβ in the nucleus and that Y48 phosphorylation inhibits the chaperone activity, expanding its mitochondrial and cytosolic functions to the nucleus.

The modification of Cyt*c* Y48 has also been studied using p-carboxymethyl-L-phenylalanine (pCMF) as an alternative phosphomimetic replacement [52]. While studies consistently reported reduced downstream caspase-3 activity, the Y48pCMF variant exhibited increased activity when tested with isolated COX and showed elevated cardiolipin peroxidase activity. Notably, despite enhanced Cyt*c*–COX activity, which is the opposite effect of Cytc Y48 phosphorylation, the overall electron transfer rate through the electron transport chain (ETC) was diminished, potentially due to impaired interaction between complex III and Cyt*c*. Thermodynamics studies indicated that in the Y48pCMF variant, the oxidized form was stabilized compared to WT [47]. Using a similar approach, it would be interesting to analyze the Y48E variant, which may cause a more perturbed structure due to the lack of the benzene ring, which is present in both WT, Y48F, and pCMF Cyt*c*.

Cyt*c* Y48 has also been studied in the context of human disease. Y48H is a disease-associated mutation of Cyt*c* that leads to mild thrombocytopenia [71], a condition characterized by a low platelet count, which can cause bleeding issues, bruising, and petechiae. Under normal cellular conditions, only a smaller fraction of the histidine pool is protonated and positively charged, which goes in the opposite direction of phosphorylation, with the phosphate group carrying a negative charge. Interestingly, the histidine mutation also reduces the respiratory rate, similar to phosphorylation, while having the opposite effect of phosphorylation on apoptotic activity, which was increased [71]. Another study reported that Y48H substitution of Cyt*c* induces a shift in the heme iron to a penta-coordinated state, which enhances its cardiolipin peroxidase activity [72]. Considering the essential roles of Cyt*c* and its overt evolutionary conservation, it is striking that one of the few known pathogenic mutations in humans of a residue that is fully conserved results only in mild thrombocytopenia.

## 5. Conclusions

**Figure 5:**
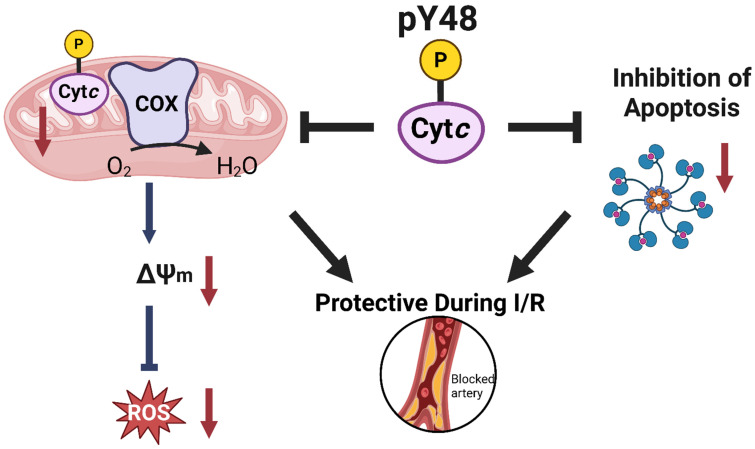
Model of Cyt*c* regulation through Y48 phosphorylation.

In conclusion, regulation of Cytc through phosphorylation affects the multiple functions of the protein, both pro-life and pro-death. Our results suggest that Cyt*c* Y48 phosphorylation results in optimal, intermediate membrane potential levels, lower ROS production, and significant protection from cell death upon OGD/R (Figure 5). However, several questions remain. For example, identification of the kinase and phosphatase mediating this (de-)phosphorylation as the next step would make it possible to target this modification. These investigations could reveal critical details surrounding potential therapeutic intervention points aimed at modulating Cyt*c* function and treating liver diseases.

## Data Availability

The original contributions presented in this study are included in the article/Supplementary Materials. Further inquiries can be directed to the corresponding author.

## Supplementary Materials

The following supporting information can be downloaded at: https://www.mdpi.com/article/10.3390/biom16050632/s1. Figure S1: (A) Non-mitochondrial respiration (*n* = 5); (B) Proton leak (*n* = 5); (C) ATP-coupled respiration (*n* = 5); (D) Maximal respiration (*n* = 5); (E) Spare respiratory capacity (*n* = 5); (F) Extracellular acidification rate (ECAR; *n* = 5). The original gel electrophoresis images of Figure 2A and the raw data for Y48 can be found in the Supplementary Materials.

## Abbreviations

Apaf-1	Apoptotic protease-activating factor-1
Cyt*c*	Cytochrome *c*
COX	Cytochrome *c* oxidase (Complex IV)
ECAR	Extracellular acidification rate
ETC	Electron transport chain
EV	Empty vector (cells without Cyt*c*)
K_3_Fe(CN)_6_	Potassium ferricyanide
ΔΨm	Mitochondrial membrane potential
Na_2_S_2_O_4_	Sodium dithionite
OCR	Oxygen consumption rate
OGD/R	Oxygen–glucose deprivation/reoxygenation
PI	Propidium iodide
PTM	Post-translational modification
ROS	Reactive oxygen species
Y48E	Cyt*c* with tyrosine 48 mutated to glutamate
Y48F	Cyt*c* with tyrosine 48 mutated to phenylalanine
WT	Wild-type (of Cyt*c*)
